# Non-dilated left ventricular cardiomyopathy in patients with chronic Chagas disease and heart failure with reduced left ventricular ejection fraction

**DOI:** 10.1093/eschf/xvag020

**Published:** 2026-01-13

**Authors:** Reinaldo B Bestetti, Augusto Cardinalli-Neto, Ana Paula Otaviano, Mauricio N Machado, Paulo R Pavarino, Marcelo A Nakazone

**Affiliations:** Department of Medicine, University of Ribeirão Preto, Ribeirão Preto city 14096-900, Brazil; Division of Postgraduation, São José do Rio Preto Medical School, São José do Rio Preto city, Brazil; Fundação Faculdade de Medicina de São José do Rio Preto, São José do Rio Preto city, Brazil; Fundação Faculdade de Medicina de São José do Rio Preto, São José do Rio Preto city, Brazil; Fundação Faculdade de Medicina de São José do Rio Preto, São José do Rio Preto city, Brazil; Department of Cardiology and Cardiovascular Surgery, São José do Rio Preto Medical School, São José do Rio Preto city, Brazil; Division of Postgraduation, São José do Rio Preto Medical School, São José do Rio Preto city, Brazil; Fundação Faculdade de Medicina de São José do Rio Preto, São José do Rio Preto city, Brazil; Department of Cardiology and Cardiovascular Surgery, São José do Rio Preto Medical School, São José do Rio Preto city, Brazil

**Keywords:** Chagas disease, Chagas cardiomyopathy, Non-dilated left ventricular cardiomyopathy, Heart failure, Inherited cardiomyopathy

## Abstract

**Introduction:**

The aim of this investigation was to establish the clinical characteristics and outcomes of patients with non-dilated left ventricular cardiomyopathy (NDLVC) secondary to chronic Chagas disease (CChD).

**Methods:**

All patients with CChD followed at our institution from January 2000 to January 2010 were included. Patients with a left ventricular ejection fraction (LVEF) <50%, a left ventricular diastolic diameter (LVDD) <55 mm, and segmental wall motion abnormalities (SWMA) on echocardiography were diagnosed with NDLVC secondary to CChD. The remaining patients had dilated cardiomyopathy (DCM) secondary to CChD.

**Results:**

Of the 215 patients, 21 (10%) had NDLVC. In this group, the mean age was 62 ± 9 years, 12 (57%) were male, the median daily dose of metoprolol was 100 (62, 5, 125) mg, the LVDD was 51 ± 29 mm, and the LVEF was 39 ± 6.4%. SWMA were found in 12 (57%) patients. Mean follow-up 50.8 ± 3.8 months, during which 9 (43%) patients died. In a Cox regression model, Beta-blocker therapy was the only independent predictor of all-cause mortality for patients with NDLVC (hazard ratio = 0.16, 95% CI 0.04-0.68; *P* = .01) Over a 60-month follow-up, Kaplan-Meier survival estimates at 12, 24, 36, 48, and 60 months, were 71%, 71%, 59%, 59%, and 59%, respectively, in patients with NDLVC, and 77%, 61%, 49%, 38%, and 30%, respectively, in those with DCM due to CChD (*P* = .04).

**Conclusions:**

Non-dilated left ventricular cardiomyopathy affects one in 10 patients with CHF due to CChD, and the 5-year survival rate is 59%.

## Graphical Abstract

**Graphical Abstract:**
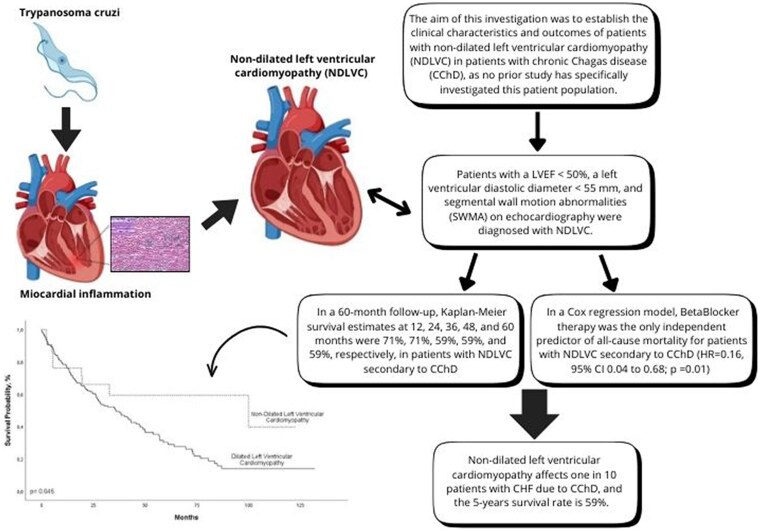

## Introduction

Non-dilated left ventricular cardiomyopathy (NDLVC) is a new clinical entity characterized by non-dilated left ventricle, regardless of the left ventricular ejection fraction (LVEF), with or without global or segmental wall motion abnormalities (SWMA), with or without myocardial scarring at cardiac magnetic resonance imaging (cMRI) in the absence of ischaemic or load conditions.^1^ A population-based study has set the prevalence of NDLVC at 0.9%–1.9%.^2^ Nevertheless, among patients with heart failure with reduced ejection fraction (HFrEF), NDLVC can be observed in about 14%–26% of patients.^3–6^ In patients with the pathogenic desmoplakin variants, about 23.4% had the NDLVC phenotype,^7^ thus suggesting that the NDLVC may be a frequent clinical condition. The incidence of NDLVC is unknown.

Several pathogenic variants have been causally associated with NDLVC, including LMNA, FLNC truncating variants, THEM 43, PLN, DSP, and RBM 20.^1^ Non-dilated left ventricular cardiomyopathy can also be found in patients without a pathogenic variant.^3–6^ In carriers of pathogenic desmoplakin variants, concomitant myocarditis can be found in 9%–40% of patients with NDLVC.^7,8^ Non-dilated left ventricular cardiomyopathy can also be observed in patients with Inflammatory Cardiomyopathy in the absence of concomitant pathogenic variants.^9^ The clinical characteristics, the clinical course, and the treatment have been established for patients with NDLVC secondary to either inherited cardiomyopathies^1,7,8^ or non-genetically based cardiomyopathies.^3,4,6^

On the other hand, Chagas disease affects about 6 million people in Latin America, and it has now been observed in the USA and Europe. The disease is caused by the protozoan *Trypanosoma cruzi*, which is transmitted to humans through the faeces of a sucking bug. Left untreated, most patients (about 60%) develop a positive serology for the disease without any evidence of heart or digestive system involvement, the so-called indeterminate stage of Chagas disease, and 20% develop Chagas cardiomyopathy up to 20 years after initial infection.^10^

Even patients in the indeterminate stage of CChD may have SWMA at left heart catheterization or echocardiography.^11,12^ In those patients with 12-lead ECG abnormalities without left ventricular systolic dysfunction (LVSD) or left ventricular diastolic dilatation (LVDD), SWMA can be observed in 48% of patients.^13^ These findings suggest that NDLVC may also be observed in many patients with CChD.

Comprehensive evaluation of NDLVC in patients with CChD and chronic heart failure (CHF) is essential for accurate risk stratification, prognostication, and opportunity for treatment in the early stage of patients with this condition, as it provides critical information on myocardial heterogeneity, contractile reserve, and arrhythmic risk. This assessment also plays a key role in clinical decision-making, as it can help to reveal candidates for advanced therapies and guides the monitoring of responses to pharmacologic and device-based interventions.

The aim of this study was to define the clinical characteristics, predictors of mortality, as well as prognosis for patients with NDLVC secondary to CChD.

## Methods

All patients routinely followed at our Outpatient Cardiomyopathy Service from January 2000 to January 2010 with the diagnosis of Chagas disease and CHF were initially considered for the investigation. As strongly recommended by the World Health Organization, we used one test of high sensitivity (indirect immunofluorescence) and one of high specificity (indirect haemagglutination) to make the diagnosis of CChD. The diagnosis of the illness was made when both tests were positive. If one test was positive and the other negative, we used the enzyme-linked immunosorbent assay to establish the diagnosis of CChD.^14^

The inclusion criteria at baseline were as follows: (i) a LVEF <50% at either two-dimensional (2-D) echocardiography or Radionuclide Ventriculography; (ii) a left ventricular diastolic diameter (LVDD) at echocardiography <55 mm, which are encompassed by that of the European Society of Cardiology.^1^ Standard 2-D echocardiography was performed within 6 months of admission to the outpatient service, and all measurements were obtained according to international recommendations at the time. Speckle-tracking echocardiography was not performed because it was unavailable during the study period. We have not included patients with normal LVEF because we did not access the data of patients with this condition, for our patients were treated in a tertiary referral centre, which is primarily dedicated to CHF.

Patients with a LVEF between 41% and 49% were diagnosed as having heart failure with mildly reduced ejection fraction (HFmrEF), whereas those with a LVEF ≤40% were diagnosed with HFrEF, according to international guidelines, and as reported elsewhere.^15^ Patients with any other disease that could induce heart disease by itself were ruled out of the investigation. Patients with electrocardiographic changes as well as those with segmental wall motion abnormalities (SWMA) at 2-D echocardiography without left ventricular dilatation were diagnosed as having NDLVC secondary to CChD. Those with a LVDD ≥55 mm were considered to have Dilated Cardiomyopathy (DCM) secondary to CChD.

At admission, patients underwent history-taking and complete physical examination. The demographic data of the cohort, including the New York Heart Association classification, was collected for each patient enrolled in the study. The systolic blood pressure, the diastolic blood pressure and the heart rate were also noted. The remaining of the work-up consisted of standard laboratory tests, 12-lead ECG, and 2-D echocardiography. Cardiac magnetic resonance imaging was not performed in any patient because it was not available during the study period.

Patients with NDLVC secondary to ChCM with HFrEF were treated with diuretics as needed, angiotensin converting enzyme inhibitors or angiotensin receptor blocking (ARB), and Beta-blockers at targeted doses or at maximal tolerated dose. Mineralocorticoid receptor antagonist (aldosterone) was given preferentially to patients in the NYHA Class III or IV, according to the results of the RALES trial,^16^ and as observed in other study enrolling patients with either isolated left ventricular dysfunction without left ventricular dilatation (NDLVC) or DCM not related to CChD.^17^ Digoxin was given to patients still symptomatic despite the treatment mentioned earlier, and amiodarone was given to patients with symptomatic premature ventricular contractions (PVC). Patients with DCM secondary to ChCM were given the same treatment.

### Statistical analysis

Continuous variables with normal distribution are presented as mean ± standard deviation, whilst those with non-normal distribution are given as median (25th, 75th percentiles). Categorical variables are given as count (percentage). The *t*-test for unpaired samples was used to compare continuous variables between groups, and the χ^2^ test to compare between-groups categorical variables.

A Cox proportional hazards model was used to establish independent predictors of all-cause mortality for patients with NDLVC secondary to CChD. One variable for each 10 deaths was entered the model to avoid the overfitting. Variables univariately associated with all-cause mortality at the *P* level <.05 were entered a multivariable logistic regression analysis with backword elimination. The proportional hazards assumption was checked by the Schoenfeld residuals test. Survival analysis between groups was carried out by the Kaplan–Meier method. Differences with *P*-values <.05 were considered statistically significant, except for the Schoenfeld test, where *P*-values >.05 for each covariate and in the global test indicated no violation of the proportional hazards assumption.

This study was carried out according to the Declaration of Helsinki. It was approved by the Human Research Ethics Committee of the Faculdade de Medicina de São José do Rio Preto (CAAE: 02716112.6.0000.5415). Because the study was retrospective, the patients were fully anonymized, and the data were obtained from standard care at the time, the requirement for individual informed consent was waived.

## Results

A total of 215 patients were included in the analysis; 21 (10%) were diagnosed with NDLVC secondary to CChD. Of these patients, 12 (57%) were male; mean age was 62 ± 9 years; 17 (80%) were in the NYHA Class I/II; 7 (33%) had been previously hospitalized; Mean heart rate was 70.7 ± 14.9 (beats per minute), and mean systolic blood pressure 108.3 ± 13.7 (mmHg). Angiotensin converting enzyme inhibitors/ARB were given to 19 (90%) patients, Beta-blocker therapy to 16 (76%), furosemide to 15 (71%), and spironolactone to 7 (33%). Mean Na potassium levels was 141.6 ± 3.6 mEq/l, mean K serum levels 4.7 ± 0.8 mEq/l, and mean creatinine serum levels 1.2 ± 0.6 mg/dl.

*Table 1* compares the clinical characteristics of patients with NDLVC and those with DCM secondary to CChD. Patients with NDLVC were older than those with DCM secondary to CChD. The proportion of patients on digoxin and spironolactone daily use was lower in patients with NDLVC in comparison with patients with DCM secondary to CChD. *Table 2* compares median daily dose of drugs given to patients with NDLVC and DCM secondary to CChD. The median daily dose of metoprolol was higher and the median daily dose of furosemide was lower in NDLVC in comparison with patients with DCM secondary to CChD. *Table 3* shows the ECG as well as the echocardiographic variables observed in patients with NDLVC and in those with DCM secondary to CChD. Atrial fibrillation (AF) was seen at 12-lead ECG in 6 (29%) patients, artificial pacemaker in 12 (57%), left anterior fascicular block in 9 (43%), right bundle branch block in 8 (38%), and PVC in 8 (38%) in patients with NDLVC. No statistical difference was observed between patients with NDLVC and those with DCM secondary to CChD regarding ECG abnormalities.

**Table 1:** Comparison of the clinical characteristics between patients with non-dilated left ventricle cardiomyopathy (***n*** = 21) and in those with dilated ventricular cardiomyopathy (***n*** = 194) secondary to Chagas cardiomyopathy

	NDLVC (*n* = 21)	DLVC (*n* = 194)
Age (years)	62 ± 9	54 ± 14*
Male	12 (57%)	131 (65%)
NYHA I/II	17 (81%)	66 (65%)
Hospitalization	7 (33%)	138 (70%)
HFmrEF	8 (38%)	28 (14%)
HFrEF	13 (62%)	166 (86%)
ACEI	19 (90%)	183 (94%)
BB	16 (76%)	102 (53%)
Digoxin	8 (38%)	158 (81%)**
Diuretics	13 (62%)	175 (90%)
Aldosterone	7 (33%)	145 (75%)**
Amiodarone	6 (29%)	75 (39%)
HR (bpm)	71 ± 15	71 ± 15
SBP (mmHg)	108.3 ± 13.7	106. 6 ± 16.2
DBP (mmHg)	69.5 ± 12	70.5 ± 10.9
Na (mEq/l)	141.6 ± 3.6	140.5 ± 5.6
K (mEq/l)	4.7 ± 0.8	4.4 ± 0.6
Creatinine (mg/dl)	1.2 ± 0.6	1.2 ± 0.6
Hb (g/l)	13.2 ± 1.4	13.4 ± 1.6

NYHA, New York Heart Association classification; HFmrEF, heart failure with mildly reduced ejection fraction; HFrEF, heart failure with reduced ejection fraction; ACEI, angiotensin converting enzyme inhibitor; ARB, angiotensin receptor block; HR, heart rate; SBP, systolic blood pressure; DBP, diastolic blood pressure

^*^ *P* = .01; **P < .005

**Table 2:** Comparison of median daily doses of drugs used in the treatment of patients with Chagas cardiomyopathy-associated non-dilated left ventricle cardiomyopathy vs Chagas cardiomyopathy-associated dilated left ventricular cardiomyopathy

	NDLVC (*n* = 21)	DLVC (*n* = 194)
Enalapril mg/day	15 (10, 20)	10 (10, 20)
Captopril mg/day	37.5 (28.13, 65.63)	75 (37.50, 75)
Losartan mg/day	50 (31.25, 87.50)	50 (25, 50)
Carvedilol mg/day	25 (12.50, 50)	25 (6.25, 50)
Metoprolol mg/day	200 (150, 200)	100 (62.50, 125)*
Digoxin mg/day	013 (0.13, 0.22)	013 (0.13, 0.25)
Spironolactone mg/day	25 (25, 25)	25 (25, 25)
Furosemide mg/day	40 (40, 80)	80 (40, 120)**
Amiodarone mg/day	200 (200, 200)	200 (200, 200)

NDLVC, non-dilated left ventricular cardiomyopathy; DLVC, dilated left ventricular cardiomyopathy.

^*^ *P* = .01; **P = .009

**Table 3:** Electrocardiographic and echocardiographic abnormalities observed in patients with non-dilated left ventricle cardiomyopathy (NDLV; ***n*** = 21) and in those with dilated left ventricle cardiomyopathy (DLV; ***n*** = 194) secondary to Chagas cardiomyopathy

	NDLVC (*n* = 21)	DLVC (*n* = 194)
ECG changes		
Atrial fibrillation	6 (29%)	52 (27%)
Left bundle branch block	2 (9%)	34 (17%)
Left anterior fascicular block	9 (43%)	75 (39%)
Right bundle branch block	8 (38%)	73 (38%)
Premature ventricular contractions	8 (38%)	90 (46%)
Pacemaker	12 (57%)	105 (54%)
Implantable cardioverter defibrillator	2 (9%)	21 (11%)
echocardiographic abnormalities		
Left ventricular diastolic diameter (mm)	51 ± 2.9	67.4 ± 7.2*
Left ventricular systolic diameter (mm)	40.6 ± 3.4	57.4 ± 7.6*
Right ventricular diameter (mm)	24.1 ± 8.3	25.4 ± 7.2
Left ventricular ejection fraction (%)	39 ± 6.4	30.5 ± 9.1*
Segmental wall motion abnormalities	12 (57%)	71 (37%)

^*^ *P* < .0005

At echocardiography, 8 (38%) patients with NDLVC secondary to CChD had HFmrEF; mean LVDD was 51 ± 2.9 mm, mean left ventricular systolic diameter 40.6 ± 3.4 mm, mean right ventricular diameter 24.1 ± 8.3 mm, and mean LVEF 39 ± 6.4%. LVWA were seen in 12 (57%) patients with this condition. In comparison with patients with DCM secondary to CChD, patients with NDLVC had lower left ventricular systolic diameter and higher LVEF. No other statistical difference was observed in patients with NDLVC in comparison with patients with DCM secondary to CChD regarding echocardiographic parameters.

**Figure 1:**
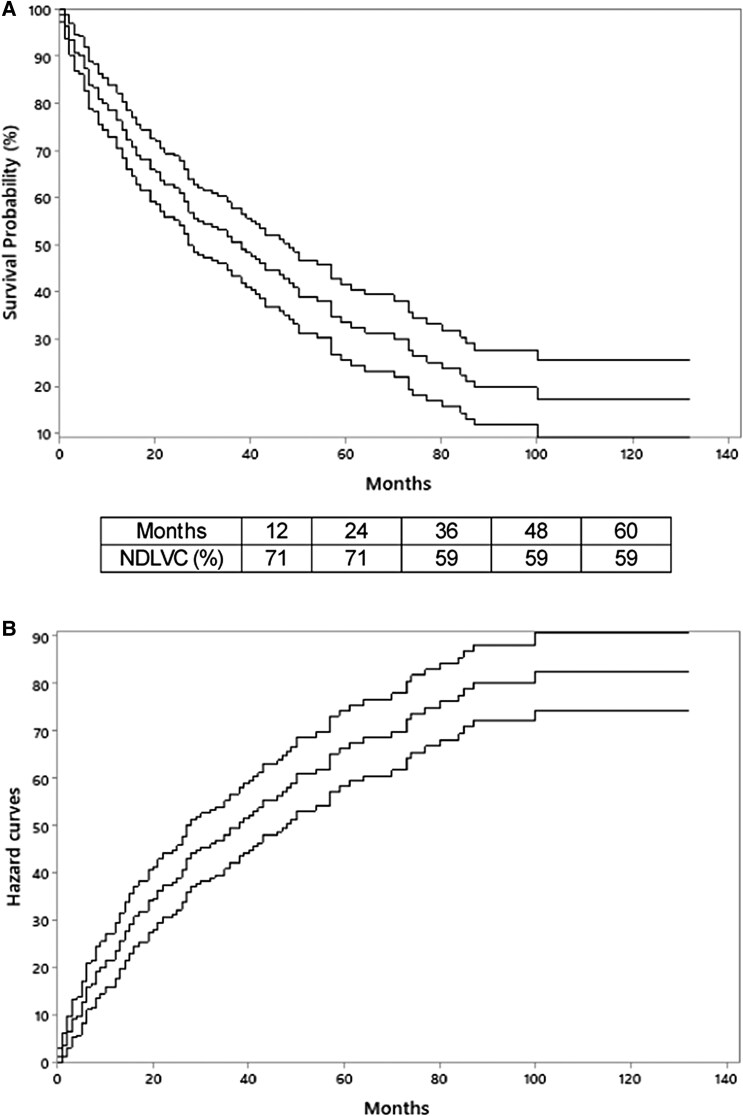
(*A*) Survival probability for patients with non-dilated left ventricular cardiomyopathy secondary to Chagas cardiomyopathy according to Kaplan–Meir curve. The curve in the middle the survival curve; the superior and inferior curves are the 95% confidence interval. (*B*) Hazard curve showing the instantaneous risk of death in patients with non-dilated left ventricular cardiomyopathy secondary to Chagas cardiomyopathy. The curve in the middle is the hazard curve. The superior and inferior curves are the 95% confidence intervals

**Figure 2:**
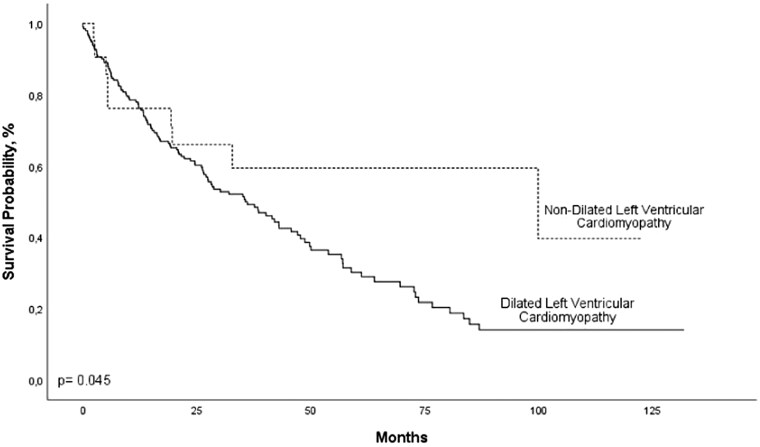
Comparison of survival between patients with non-dilated left ventricular cardiomyopathy and those with dilated left ventricular cardiomyopathy secondary to Chagas cardiomyopathy

Mean follow-up of patients with NDLVC secondary to CChD was 50.8 ± 3.8 months; 9 (43%) patients died. The survival rates at 12, 24, 36, 48, and 60 months of follow-up was 71%, 71%, 59%, 59%, and 59%, respectively, in patients with NDLVC secondary to CChD (*Figure 1A*). *Figure 1B* depicts a hazard curve showing the instantaneous risk of death for patients with NDLVC secondary to CChD. BB therapy was the only independent predictor of all-cause mortality for patients with NDLVC secondary to CChD (hazard ratio = 0.16, 95% confidence interval 0.04–0.68; *P* = .01) in a Cox regression model. No violation of the proportional hazards assumption was found for any of the covariates tested or in the global test, as the *P*-values were >0.05 in all cases. Mean follow-up was 30.5 ± 28 in patients with DCM secondary to CChD. All-cause mortality was detected in 117 (60%) of 194 patients with this condition. Kaplan–Meier survival estimates were 77%, 61%, 49%, 38%, and 30% at 12, 24, 36, 48, and 60 months of follow-up, respectively in patients with DCM secondary to CChD (*P* = .04 in comparison with NDLVC secondary to CChD). *Figure 2* illustrates these data. As can be seen, survival in patients with NDLVC secondary to CChD was higher compared with that of patients with DCM secondary to CChD.

## Discussion

As far as we know, this is the first study dealing with NDLVC in the setting of CChD. This study shows that NDLVC can be found in about 10% of patients with CHF secondary to CChD. Moreover, about 38% of such patients had HFmrEF. The clinical characteristics as well as the electrocardiographic abnormalities in patients with NDLVC are similar to those with DCM secondary to CChD, whereas the LVEF is higher in the former in comparison with the latter. Long-term outcome of patients with NDLVC secondary to CChD appears to be poorer than that observed in NDLVC not associated with CChD in a 5-year follow-up.^17^ These findings suggest that the new definition of NDLVC established by the European Society of Cardiology^1^ can also encompass patients with CChD with CHF.

The prevalence of NDLVC secondary to CChD observed in this investigation (10%) is similar to that found in patients with inherited cardiomyopathy^7^ or no genetic-based cardiomyopathy^3–6^ treated in referral centres, which varies from 14% to 26%. The prevalence NDLVC secondary to CChD observed in our study is consistent with the studies performed in those with NDLVC of other aetiologies.

A retrospective view of previous articles on CChD allows us to estimate the epidemiology concerning NDLVC in patients with this condition. Espinosa et al. have observed that 12 (12%) out of 107 patients of a population-based cohort of CChD patients with normal resting ECG had SWMA by left ventricular angiography without left ventricular dilatation consistent with NDLVC.^11^ Ortiz *et al*.^12^ studied 30 patients in the indeterminate stage of CChD, and they observed that 6 (20%) of such patients had normal LVEF and SWMA at 2-D echocardiography also consistent with NDLVC. Câmara observed SWMA in 19 out of 39 (48%) patients with abnormal 12-leading ECG and no left ventricular dilatation, especially in the apex region.^13^ Our demographic data are consistent with previous studies on CChD, which showed that the prevalence of NDLVC in such patients can vary from 12% to 48% depending on the stage of CChD.

The clinical characteristics of patients with NDLVC secondary to CChD may be similar to those observed in patients with genetically based cardiomyopathies. In patients with DSP cardiomyopathy, the annual incidence of malignant ventricular arrhythmia and hospitalization in those with a LVEF <50%, the annual incidence of malignant ventricular arrhythmias and HFrEF hospitalization are 3.6% and 4.4%, respectively.^7^ About 68%–78% of such patients are found to be in the New York Heart Association (NYHA) Class I/II, and 50% have been hospitalized for heart failure treatment in the precedent year.^3,4^

In our study, the incidence of Implantable Cardioverter Defibrillator, which has been used to treat malignant ventricular arrhythmia, can be roughly estimated to be 2, 1% yearly; the prevalence of previous hospitalization and the NYHA Class I/II were 33% and 81%, respectively. This suggests that the clinical picture of NDLVC secondary to CChD resembles that of NDLVC unrelated to either CChD or genetically based cardiomyopathies.^4^

The ECG abnormalities observed in our patients are remarkable for a high frequency of intraventricular conduction disturbances, advanced AV block treated with pacemaker, AF, and PVC. Such electrocardiographic abnormalities have been observed in inherited cardiomyopathies as well.^1^ Prolonged PR and AV blocks are characteristics of genetically-determined neuromuscular disorders, whereas conduction disease and arrhythmias are also observed in PLN and FLNC truncating variants.^1^ In patients harbouring a DSP gene variant, PVC, Non-sustained ventricular tachycardia, and sustained Ventricular Tachycardia can be observed in 60%, 60%, and 80% of patients, respectively.^8^ Prolonged PR interval, AF, and PVC are usually seen in the resting ECG of patients with NDLVC secondary to laminopathies^1^; complete AV block can also be detected in such patients.^9^ Except for the high frequency of malignant ventricular arrhythmia observed in DSP cardiomyopathy, the ECG abnormalities observed in patients with NDLVC secondary to CChD are similar to those found in some types of NDLVC secondary to other cardiomyopathies.

Sixty-seven per cent of our patients with NDLVC secondary to CChD had SWMA at echocardiography, which was similar to that found in those with DCM secondary to CChD. The importance of the detection of SWMA in the setting of CChD is still controversial regarding its impact on the clinical course of the disease. In a mean follow-up of 4.9 years, Espinosa *et al*.^11^ observed that 4 out of 12 (33%) patients with NDLVC with normal resting ECG and SWMA at left ventricular angiography progressed to overt DCM. Pazin-Filho *et al*. studied 14 patients with CChD with SWMA at 2-D echocardiography and 45 patients without such abnormalities and no LVSD. In a mean 4.6 years follow-up, they observed that LVSD appeared in 71.4% of patients with SWMA and in 22.2% of patients with no SWMA *P* < .005.^18^ Conversely, Viotti *et al*. enrolled 849 patients with NDLVC secondary to CChD in a mean follow-up of 9.9 years; 13% of them in the indeterminate stage and 33% with abnormal resting ECG and no left ventricular dilatation had NDLVC. The presence of SWMA at echocardiography were not found to be a risk factor for mortality in such patients.^19^

The presence of NDLVC secondary to CChD can be associated with complex PVC at 24-h Holter monitoring. In the study by Barros *et al*., SWMA were found in 12 out of 37 (28.7%) patients with NDLVC secondary to CChD without LVSD, and they were associated with complex PVC (Grade IV-A of Lown classification).^20^ Pedrosa *et al*.^21^ showed that 25% of patients with NDLVC secondary to CChD characterized by normal 12-lead ECG, normal LVEF and SWMA at echocardiography exhibited PVC (Grade III of Lown classification). The impact of such arrhythmia on the prognosis of patients with NDLVC secondary to CChD remains obscure.

Beta-blocker therapy was associated with a reduced risk of all-cause mortality for patients with NDLVC secondary to CChD. Beta-blocker therapy has been shown to be a predictor of all-cause mortality for patients with DCM secondary to CChD.^15,22^ Furthermore, Beta-blocker therapy has been suggested to have a favourable effect on the clinical course of patients with CChD with HFrEF.^23,24^ Experimentally, chronic *T. cruzi* infection resembles a catecholamine cardiomyopathy,^25,26^ and Beta-blocker therapy also improves the natural history of *T. cruzi* infected rats.^27^ The fact that Beta-blocker therapy was a predictor of all-cause mortality for NDLCV secondary to CChD is consistent with previous studies carried out on patients with CChD. Although the Schoenfeld test revealed no violation of the proportional hazards assumption for any of the covariates tested or in the global test, the possibility of residual confounding still exists. Predictors of all-cause mortality and the clinical course of patients with NDLVC secondary to CChD could differ in another context—for example, if patients with NDLVC without LVSD had been included.

Beta-blocker therapy has been recommended by the ESC guidelines for the management of cardiomyopathies to treat patients with NDLVC not associated with CChD, as a means to prevent the progression of left ventricular dilatation and dysfunction.^1^ Our study suggests that beta-blocker therapy may also be useful for patients with NDLVC secondary to CChD with HFrEF or HFmrEF. Whether beta-blocker therapy benefits patients with NDLVC and normal LEVF remains to be determined.

This investigation demonstrated high 5-year mortality among patients with NDLVC secondary to CChD. This high mortality is notable, given that some studies suggest that mortality in patients with CChD without LVSD can be similar to the general population,^11^ and SWMA *per se* has not been identified as a risk factor for mortality.^19^ Our cohort showed that LVSD overshadows the prognosis of patients with NDLVC secondary to CChD. Approximately 38% of our patients had HFmrEF, a clinical condition with a dismal prognosis,^15^ while the remaining patients had DCM secondary to CChD with HFrEF, which also carries a worse prognosis than that seen in other cardiomyopathies,^28,29^ with heart transplantation being the only option in advanced stages.^30^

There was a discrepancy between the mild clinical symptoms and the unfavourable prognosis of patients with NDLVC secondary to CChD. This may be partly explained by the fact that almost half of the patients were included in the cohort following a previous hospitalization for compensation of acute heart failure decompensation. Such a discrepancy might reflect the relentless poor prognosis of patients with this condition, especially those with CHF.^24,28–30^ The low proportion of patients receiving mineralocorticoid antagonists, as observed in other studies of the time,^17^ likely influenced by the results of the RALES trial, may have also contributed. These findings may account, at least in part, for the observed discrepancy between symptomatology and clinical prognosis observed in patients with NDLVC secondary to CChD.

The poor prognosis of patients with NDLVC secondary to CChD may be ascribed to the pathogenesis of chronic cardiomyopathy, which is the consequence of the interplay of autoimmunity, autonomic destruction, and microvascular coronary artery disease,^31^ leading to intracardiac sympathetic overactivity,^32^ mononuclear cell infiltrate along with reparative fibrosis throughout the myocardium.^33^ In patients with acute myocarditis, *desmoplakin* and *titin* truncating variants can be found in approximately 1.3% and 6.6% of cases, respectively.^34^ In patients with alcoholic cardiomyopathy, *titin* truncating variants are observed in about 12% of cases,^35^ similar to the frequency reported in peripartum cardiomyopathy^36^ and in cancer therapy-induced cardiomyopathy.^37^ Polymorphisms in certain genes, such as *MICA-129* and *KIR*, may be involved in the pathogenesis of HFrEF due to CChD.^38^ Whether an overlap between genetic predisposition and myocarditis also occurs in patients with NDLVC secondary to CChD remains to be determined.

### Study limitations

The major limitation of this study is the lack of cMRI data, which would be important to reveal and to quantify the amount of myocardial fibrosis. This would be important to improve prognostic indices, as it has previously been shown,^39^ but this exam was not available at our institution during the study period. Future studies using cMRI will be necessary to fully characterize NDLVC and to improve prognostication in the setting of CChD with CHF. Another important limitation of this investigation is its retrospective design which carries all the inherent biases typical of such studies, especially the selection bias that may have arisen from the long period of enrolment. Prospective studies are needed to confirm our data.

The small sample of patients with NDLVC represents another limitation of the study, as it constitutes a potential source of bias. Another limitation is that we did not include patients with NDLVC secondary to CChD and normal LVEF, whose prognosis may differ from that observed in our investigation. In the study by Espinosa *et al*.^11^ no patient with NDLVC secondary to CChD and normal LVEF died during a 65 ± 26-month follow-up. Similarly, Viotti *et al*.^19^ reported no deaths in patients with this condition over a 9.9-year follow-up. This selection bias reduces the generalizability of our findings. Despite these caveats, our data is useful in demonstrating that the European Society of Cardiology definition of NDLVC can also encompass patients with CChD.

## Conclusions

Non-dilated left ventricle cardiomyopathy affects about 10% of patients with CHF due to ChCM. Their clinical profile resembles that of patients with DCM secondary to CChD and genetically based cardiomyopathies. The 5-year survival rate is 59%.

## Declarations

### Pre-registered Clinical Trial Number

None supplied.
